# Learning contexts at Two UK medical schools: A comparative study using mixed methods

**DOI:** 10.1186/1756-0500-5-153

**Published:** 2012-03-19

**Authors:** Andrew Grant, Paul Kinnersley, Max Field

**Affiliations:** 1 Dept of Primary Care & Public Health, Cardiff University, Cardiff, UK; 2 School of Medicine, Glasgow University, Glasgow, UK; 3 Institute of Medical Education, Cardiff University, UGT 162A, University Hospital for Wales, Cardiff, CF14 4XN

## Abstract

**Introduction:**

The context in which learning takes place exerts a powerful effect on the approach learners take to their work. In some instances learners will be forced by the nature of a task to adopt a less-favoured approach.

In this study, we used a combination of qualitative and quantitative methods to compare the effect of context on learning at different UK medical schools. We compared schools with conventional, and problem-based curricula.

**Method:**

We had collected data from 30 interviews with third year medical students in one UK medical school with a conventional, lecture-based curriculum in relation to a previous study. The interview guide had explored effects of context and approach to learning. We used the same guide to interview 6 students in another UK medical school with a problem-based curriculum.

We then put together a pack of validated questionnaires, which measured the phenomena that had emerged in the interviews. In particular we selected questionnaires which measured the criteria on which students from the different schools appeared to demonstrate greatest variance.

**Results:**

There were two areas where students from schools with differing curricula differed - basic learning activity and assessment. Students at the lecture-based school attended lectures where they received information while students at the Problem-based school attended tutorials where they stimulated prior knowledge and identified new learning objectives. Progress -testing at the problem-based school helped students gain a sense of accumulating a body of knowledge needed for their life in medicine while students' at the lecture-based school directed their learning towards passing the next set of exams.

The findings from quantitative, questionnaire data correlated with the interview findings. They showed that students at a school with a PBL curriculum scored significantly higher for reflection in learning, self-efficacy in self-directed learning and for deep approach to learning.

**Conclusion:**

We set out to determine whether students at different medical schools approach their learning differently. We have succeeded in demonstrating that this is the case.

The differences that we detected in learning context and approaches to learning in medical students at the two schools predict that learning at the non PBL school is likely to be via a surface approach and not integrated. These differences have major implications for the outcomes of medical student learning at the two schools in terms of accessibility and sustainability of learning.

## Introduction

### Effect of context on learning

The context in which learning takes place exerts a powerful effect on both the process and the outcomes of learning [1]. It can influence students' interest in and motivation towards their studies [2]. Research shows that students' interest, attitudes to studying and learning approach are influenced by experience [2]. Research also shows that learners concept of knowledge and learning are acquired through experience and that this becomes more sophisticated over time [3].

Learning environments where students perceive that teaching is good and where they enjoy freedom in their learning foster a deep approach to learning. Learning environments characterised by a "heavy workload" and "lack of freedom" conversely lead to students adopting a surface approach [4]. A task that demands that the learner absorbs an overwhelming volume of knowledge will steer them towards a surface approach [5].

Learning styles and approaches are not fixed characteristics. The same student will take a different approach to consecutive learning tasks if that is what those tasks demand. Lindblom-Ylänne & Lonka [6] in their study with senior medical students describe "Dissonant Study Orchestration". This is where students have a desire to understand rather than memorise course material but feel forced by content volume and assessments to take a surface approach. Dissonant study orchestration is associated with disillusionment and de-motivation.

In order that they guide their students towards a deep approach to learning curriculum leaders are advised to plan the learning environment for their students separately from course content and assessments [1].

### Hidden curriculum

As well as from their course materials and learning activities, students gain a great deal of information about their course, the approach required and how to survive from other students. They are most likely to gain this information from their peers and students in years above [7]. The term "Hidden curriculum" has been used to describe this unofficial, handed down information, which is defined the as "The set of influences that function at the level of organisational structure and culture including, for example, implicit rules to survive the institution such as customs, rituals, and taken for granted aspects." [7]. It is easy to see that this set of influences might affect the approach students take to their learning.

## Background

When analysing data for a previous study [8] it became clear that the learning context at the school where the study was being carried out was exerting a major influence on students' learning practice and whether they engaged with reflective learning activities. They described the effect of the volume of material to be learned and the form of assessments on their learning. They also described clearly the effects of the hidden curriculum [8,9]. During analysis of the data and discussion of the emerging thematic framework the question was raised whether students at different medical schools would have responded in the same way to interviews based on the same schedule (see Appendix).

### Research question

This study, therefore, used a combination of qualitative and quantitative methods to examine the following hypotheses.

1. Students at different UK medical schools will respond differently to interviews exploring contextual effects on their learning and their conceptualisation of learning".

2. Students at different UK medical schools with different curricula will score differently on psychometric instruments measuring reflection in learning, self-efficacy in self-directed learning and in approach to learning.

## Method

### Mixed method study

This study was carried out in two stages using different methodologies. Firstly in the qualitative phase interview data from students at two UK medical schools with different curricula were compared with a particular focus on approaches to learning and conceptual understanding of learning. In the second, quantitative phase we tested our findings from the qualitative phase with much larger numbers of respondents using a pack of validated questionnaires.

### Ethical approval

At the time that this research was carried out no official mechanism was in place covering research involving medical students. The Local Research Ethics committee were consulted and we were informed that this study did not need their approval. The study proposal was submitted to the student representative body and their approval was given.

### Qualitative phase

#### Qualitative phase; interviews

We had already collected data from one to one interviews with 30 third-year medical students at one UK medical school with a lecture-based curriculum (School 1) [8]. The interview guide asked for information on the effect of context on students' learning in general and on their choice whether or not to participate in a reflective learning study (see Appendix).

We interviewed students at a second UK medical school using the portion of the interview schedule that related directly to curriculum, hidden curriculum and other institutional factors. We omitted the parts of the schedule relating to the original reflective learning study as these were not relevant to this study [8].

We selected the second school (School 2) because; by being problem-based its curriculum was demonstrably different and had a different theoretical basis to the curriculum at school 1. Assessment at school 2 included the use of progress testing. In progress testing, students in the third, fourth and fifth years all sit the same multiple-choice paper twice a year. The pass mark increases for each year.

#### Qualitative phase; subjects

We interviewed six students from school 2. Convenience sampling was used to recruit six third-year students. We compared our findings from these interviews with data collected from one to one interviews with 30 participants in the reflective learning study at school 1. All participants consented to take part in the study.

#### Data analysis

Interviews were carried out using the interview guide (see Appendix). They were audio recorded and recordings were transcribed. N6 software was used to support data coding [10]. Two researchers carried out initial coding independently. They then met to discuss and compare the emerging thematic framework and the N6 software was used to develop themes (nodes) and subthemes. This framework was then used to further code the data. The note facility of N6 was utilised to make timed records within the framework for future reference.

#### Qualitative phase; validation of analysis

An independent expert in qualitative research methodology examined six interview transcripts (approximately one sixth) developing an independent list of themes. No themes were revealed that were not already included in the framework but some themes were missing from her list. We undertook participant validation by sending a summary of findings to a sample of the study participants who were asked to validate whether our findings were, in their view representative of their learning and the effects of context upon it. None of the five students who replied contested any part of the précis of findings that was circulated.

### Quantitative phase

#### Quantitative phase; subjects

It did not prove possible to include students in school 2 in the quantitative phase of this study. We were able, however, to involve students in a third UK medical school (school 3) in this phase. Like school 2, school 3 has a problem-based curriculum, demonstrably different from the curriculum in school 1.

All final-year students at schools 1 and 3 were invited to participate in this phase. Final-year students were chosen as those who had longest exposure to the respective learning strategies at each of the two schools. All participants were given written information about the study and the questionnaire and all gave consent to participate.

#### Validated instruments

The questionnaire pack was designed to test the principal themes that came out of the analysis of the qualitative data. It was based on three validated instruments.

• The reflective learning scale is designed specifically to measure reflective activity in medical students [11,12].

• The approaches to learning and studying inventory (ALSI) measures approach to learning on a number of subscales [13,14]. Unlike the Short Inventory of approaches to learning used by Coles it specifically measures deep and surface approaches to learning. Both are, however, based on the same body of theoretical knowledge.

• The self-efficacy in self-directed learning scale [15] was adapted to measure self-directedness of learning in medical students.

#### Quantitative phase; response and respondents

The questionnaire was completed by 154 of 241 (64%) students at the lecture-based school and by 237 out of 253 (94%) at the problem-based school. We asked them, opportunistically, to complete and return the questionnaire when the whole cohort was gathered for lectures.

There were no statistically significant differences in mean age (p = 0.10, Independent samples t test) or gender (p = 0.246, Chi-squared test). A higher number of problem-based students (9% compare to 3%) had acquired a university degree prior to admission. Conversely greater number of lecture-based students had taken a gap year (23% compared to 8%).

## Results

### Qualitative results

#### Key to participants

RLS*n *= student from School 1. M*n *= student from School 2.

Two themes emerged which represented areas of influence on students' learning. These were;

1. Basic learning mechanisms and

2. Assessment.

Volume of material to be learned was an issue for students at both schools although viewed quite differently.

#### Basic learning mechanisms

The basic learning activity at two schools was different comprising lectures at school 1 and Problem-Based Learning (PBL) tutorials at school 2.

Students' perception of the purpose of these activities was quite different. School 1 students perceived that the most important function of the lecture was the transfer of information from lecturer to student.

*A lecture is obviously the easiest way to get it across to you but you just write because, there is a lot of written information that you have to get down and quite often it goes in this ear and straight out onto the paper rather than actually going into your head at all*.

RLS 20

*It depends on the style of the lecture. I can't do a thing if all you're doing is hurting your hand to write as much as you can from the slides, you can't, you just have to, write, I just find those completely useless, and there's just too much of an information overload*.

RLS 1

*I remember from lectures I've found interesting, but on a Monday when you've got five or six lectures and it's one after the other and it's the same topic you tend to switch off, sitting down taking notes, so no, I don't remember much about them*.

RLS 3

Some school 1 students described good lecturers who helped them to understand underlying concepts.

*So just somebody who sits down and says these are the five essential points that I want them to take away from this lecture and these are the ones I'm going to focus on... Somebody who has thought about the topic they are going to talk about and has thought this is the essential information that they need to know*.

RLS 19

Students from school 2 described quite a different process when they discussed the week's "case" in a small group, thinking about and sharing what they new about the subject matter. Each case was discussed twice (opening and closing).

*... I think from both the opening and the closing in the discussion which when I read the opening there will be 20 things that I don't know anything about, but there maybe one thing that I do know about and you just sit there and even talking almost like the blind leading the blind there will always be something someone knows a bit about and somebody else knows someone who had something, and by the end of the opening I understand a whole lot more about the case. Without going away and reading one thing about it, you raise questions just by talking about it and people will be able to answer it, and so I can learn a huge amount by just opening it, and then you go away obviously and study it and then come back. And then coming back you close, almost raising as many questions as opening*...

M1

#### Workload & assessment

Students at both institutions perceived themselves as having a vast amount to learn. However methods of assessment were quite different and this framed the learning requirements differently. At school 1 students perceived the need to learn the content of all their lectures so that they could reproduce this at the next exam.

*We've got an exam in a fortnight I think it is and that's got something like 83 lectures worth of material in it*.

RLS 18

Interviewer: How much of your learning is in response to the prospect of exams?

*Student: Most of it (laughs) I don't think it should be that way. I think you should be learning, sort of to gain knowledge on the wards and for the clinical experience but at the end of the day it's the exams which make me work I think*.

RLS 18

*Yeah, thinking beyond qualifying and also I suppose thinking exams, there's nothing like an exam to make you have to learn about something, but it's not a particularly satisfying way of learning... but at the end of the day if you've got to pass an exam and there's going to be an exam you need to know about*.

RLS 14

The format of assessments also was seen as driving rote memorisation.

*This kind of EMQ*^1 ^*thing where you don't really get to show what you understand about a topic, it's more what random facts you can remember, you're going to get a big list, and you're going to have to put the right box next to the right thing, so a lot of it was just learning lists*.

RLS1

In contrast to this students in school 2 (where knowledge was assessed using a twice-yearly Progress Test) students, while being aware of the large volume of material to be learned, orientated their learning towards the goal of becoming a doctor as well as passing medical school exams.

*There's such a huge amount that at some point you need to cover, but because you can't cover it all in such a short space of time for the exam at the end of the year, you have to cover it all by the end of your 5 years*...

M1

*... emphasis is on what is important from the learning has changed from 'I need to pass an exam in May' to 'this is what I need for the rest of my life. I have to know this for good'*.

M3

For some school 2 students revision was seen as marginal or unnecessary but was at the heart of school 1 students' learning practice where they described techniques of rehearsal and memorisation.

... you are not meant to revise for the progress test, which is the written exam.... for someone like myself, who is not fantastic at remembering things I have to sit down and read it again, and to do that I have to revise 

M2

*When I'm revising, I take my lecture notes, which I write in pencil. The aim is to write them up sometime before I start revising but usually it's part of my revision process, go to my lecture notes and write them up neatly. Then I might, I quite like making revision cards and it's not so much I have to go back to revision cards and learn them, it's another way of writing things out. Sometimes I might just use a notebook and each page in a notebook is a lecture or something like that so a lot of my learning process is by writing*.

RLS 17

*I try to make separate notes, the notes that we take down during lectures are a bit haphazard and untidy, so I've tried to make separate notes and maybe integrate some stuff that I've read up in books*.

RLS 3

*I use recall method where I read, I study what we've done before and I try to recall it by putting the book aside and seeing if I can recall the facts*.

RLS 12

#### Culture and hidden curriculum

In carrying out the interviews and examining the data a difference emerged in students' self-confidence as learners and in their confidence in their undergraduate programme. The way in which students described third year to students junior to them varied between the two schools.

*Yes, most of them said that you're going to like 3 rd year because it's not like your 1st and 2nd year and you'll enjoy it very much*.

M6

I think if someone from 2nd year were to ask me yes I would say that 3 rd year has been wonderful 

M6

In contrast exams appeared to cast a shadow over enjoyment of third year students in school 1.

*If every student was able to do the end of year exams or had the feeling that they would pass then the third year would become the best year in medicine. It's the end of year exams that makes people say that, [it is hard] not the content of the work*.

RLS 12

In describing their Problem-based learning tutorials students described how they freely shared what they knew with their peers and felt that they gained from this. In contrast students at the lecture-based school were wary about admitting to how much study they had done.

*Everyone seems not to like someone who works hard and shuns [them] and calls them brilliant whereas you know the person might just be working hard and that's why he's producing results and there's a lot of lying that 'we didn't do any work' when they did work*.

RLS 12

Differences also existed in how students perceived their course and its ability to prepare them for life as a doctor.

*I think the way the our course is run I think it's good for the future because it's making me think on my feet and rather than just sitting in lectures and being quite passive, it's making me go away and work things out for yourself and find ways in which to learn, like going to clinic and things and so I think it's making you into a more well-rounded doctor for the future*.

M4

The first study in school 1 was set up to examine the effects of reflective learning techniques in undergraduate medical students. For some students at least this revealed a version of dissonant study orchestration whereby they were pushed, by their assessments, towards a surface learning approach.

*... You'd have to change the way we're examined- [which] is not conducive to people who learn reflectively, because it's not about learning lists, it's about learning concepts, it's about learning ideas, and It's about learning general principles, it's not about long lists, as I understand it*.

RLS1

#### Confidence

School 2 students demonstrated an air of confidence in their learning. This appeared, at least in part, to result from a confidence that they knew what they needed to know and knew that they knew it. This resulted in differing stress levels at exam times.

*When I did [the] OSCE in July usually immediately before my exams I'm so stressed out and panicking cramming as much information as I can into my head which for the progress test I did reading anything and everything I could get my hands on. When it came to the OSCE for the week before, you think 'it's a week before the exam I'm going to focus and practise' and after 2 or 3 days you got to the stage where I was sitting there and we were in groups with people and you're practising and you're thinking well I can do this, I know this, I understand this, I've been doing this all year. I don't need to sit and memorise this and learn this now until it actually got to the stage on the day of the OSCE and I was sitting in my first station, (a rest station,) so I was just sitting there, which normally you would think could be quite an intense experience just making everything worse and I said there's nothing they can throw at me today that I can't do. I know that I can do it*.

M1

### Quantitative results

#### Internal consistency

The internal consistency between all questionnaire items was 0.881 (Cronbach's alpha). The Internal consistency of individual questionnaire components were RLS 0.905, Self-efficacy for self-directed learning 0.855 and LSQ 0.793 (Cronbach's alpha).

#### Comparison of means

Mean scores for items on all three instruments were compared between final year students at schools 1 and 3 using t test for independent means.

#### Quantitative phase; findings

Students from school 3 scored significantly higher on the reflective learning scale than students from school 1. Students from School 3 also scored higher on the self-efficacy in self-reflective learning scale. Although there was no significant difference on scores for the ALSI students from school 3 scored higher on the deep learning and organisation of study subscales (see Table 1).

**Table 1:** Comparison of mean scores on the Reflective Learning Scale, Self-Efficacy in Self-directed learning scale and ALSI

Scale	School 1	School 2	Mean difference	Significance (2 tailed)
			
	mean	Mean		
*Reflective Learning Scale*	58.55	63.35	- 4.80	.001*



*Self-Efficacy in Self-Directed Learning*	50.69	54.50	-3,81	.001*



*Deep approach*	27.32	28.85	-1.53	.006*

*Surface approach*	22.49	21.71	0.78	.192

Monitoring study	27.23	27.54	-0.30	.564

Organisation of study	18.10	19.86	-1.76	.001*

Effort management	20.90	21.62	-0.72	.113

(t test for independent means)

## Discussion

By combining in-depth interviews with validated questionnaires it has been possible to triangulate findings to address the two hypotheses.

1. Students at different UK medical schools will respond differently to interviews exploring contextual effects on their learning and their conceptualisation of learning.

2. Students at different UK medical schools with different curricula will score differently on psychometric instruments measuring reflection in learning, self-efficacy in self-directed learning and in approach to learning.

Quantitative methods in the second part of this study allowed a larger number of medical students to provide opinions. However, data from student interviews in the qualitative phase, have clarified some of the reasons that students attribute to the differing learning practices. The combination of the two approaches, support suggestions that students in problem-based curricula are more likely to be self-directed in their learning, to have a more holistic approach to learning and a greater sense of self-efficacy.

### Contrasting approaches to learning and assessment

The qualitative data demonstrate a difference in learning practice, learning orientation and in perceived aims of learning. In school 1, students attend lectures, which they perceived as the main source of information. They then sought to learn that information, often by rote using rehearsal techniques. This learning activity was designed to prepare the student for their next set of exams. Some students voiced the wish that their learning was for understanding or for preparation for medical practice but the need to pass the next exam was more immediate, demonstrating dissonant study orchestration [6].

Compared to this, school 2 students' process of learning was quite different. Attending PBL tutorials was seen as being about stimulating what they already knew in relation to the case of the week then determining what needed to be learned. Although they helped each other through discussion, the tutorial was not, primarily, a source of information. It was where they decided what they needed to learn and find out elsewhere to enhance their understanding.

Assessment at the two schools also exerted different influences on learning. The progress test gave the message to students at school 2 that a gradual acquisition of knowledge was occurring in preparation for their future in medicine. Students were aware that this did involve learning a significant amount but that it could be done gradually, building on prior experience. Students at school 1 on the other hand, saw their exams as requiring the reproduction of isolated facts, which, in turn, rewarded rote learning rather than acquisition of understanding.

The quantitative data support these differences in approach. Students at the problem-based school were more reflective in their learning. In qualitative analysis, these students discuss thinking about what they know already, developing their own learning objectives along with the PBL group, so it is perhaps not surprising that their learning practice involves more reflection.

Similarly, the student's descriptions of their learning processes imply that PBL students' decide for themselves what they need to learn as well as what approach they will take. Hence, it is not difficult to see that they might become more self-efficacious as self-directed learners. This in comparison to students who write down what their lecturers say then try to learn it by rote.

Students who determine learning outcomes for themselves and then take responsibility for finding information they need to learn are also more likely to take a deep approach to learning. Using curiosity aroused by PBL cases and discussion with their peer group, these students perceive that they were more likely to have in interest in understanding and not merely having to learn to reproduce subject matter. In addition, school 3 students scored significantly higher for organisation of study, which is recognised as a marker for strategic learning (the ability to learn by surface or deep approach depending on the demands of a particular task). Students in school 1 described a less flexible pattern where they learned in preparation for exams while students in school 3 showed an understanding that they were learning for their future in medicine rather than any one set of exams. Students also demonstrated an aptitude for identifying for themselves what they needed to learn. In school 3, students described themselves as more strategic, organised learners than those in school 1. These findings predict that school 1 students were more likely to adopt a surface approach to their learning, had a lower sense of self-efficacy as learners and were less likely to engage in reflective learning techniques. These parameters predict that they will probably learn in a way that is less likely to result in a deeper level of understanding and will be less likely to integrate their learning [1,8,16]. As well as these stark contrasts in learning practice the students written comments also highlight different levels of confidence in themselves as learners and in the learning strategies at the two schools.

### Limitations

The qualitative study was carried out alongside another larger study [8,9]. It is possible that the six students from school 2 may have been a self-selected group of students. Their observations are, however, consistent with those in another study where 44 year-two students on a problem-based undergraduate medical course were interviewed [17]. Hence it is unlikely that they are unrepresentative of their cohort.

The findings have concentrated on learning style as a cause of difference in approach to study at the two schools as attributed by the respondents (students at the two schools). Clearly these will not be the only differences, but it was beyond the scope of this study to determine all the areas of difference between the two schools and the contribution those areas of difference made to variations in approach to learning.

In addition, both phases of the study could not be undertaken with students from the same medical schools for administrative reasons. Hence, other UK medical schools with a problem based curricula were approached to continue that part of the study. While this means that data do not reflect a direct comparison, it has resulted in being able to show complementary data from three UK medical schools collected using different methodologies demonstrating that students at medical schools with different curricula do take a different basic approach to their learning, This has implications for curriculum design in the future.

### Summary

Data from qualitative and quantitative analysis show that students at different medical schools adopt different approaches to their learning during undergraduate training. Students attribute these differences to the curriculum design and examination style at their school, and suggest that this could have implications for life long learning.

## End Notes

^1 ^Extended matching question

^2 ^This will have formed part of a larger interview guide for students participating in the reflective learning study in school 1

## Competing interests

The authors declare that they have no competing interests.

## Authors' contributions

AG designed the study, put together the questionnaire pack, and analysed the data. PK supervised the study design. MF supervised data collection. All authors read and approved the final manuscript.

## Appendix

Interview guide^2^

1. **Introduction **and explanation of study (brief)

2. **Reassure **about confidentiality & lack of need to please the interviewer

3. **Confirm **consent to record the interview.

4. **General learning and background**

How they set about their learning? *(How had they set about their learning i.e. before the 3^rd ^year or at the beginning of the third year before the study started? You are trying to get them to describe their approach to learning at some point before the study)*

**Probe **lectures, personal study, work with peer group.

### The college culture

How do they think other students approach learning? If differently why? How helpful have they found other students to be?

Had they themselves changed the way they learned since being in the college?

How helpful have they found the staff in advising how to learn rather than what to learn?

What motivates them to learn?

### The workload in the 3^rd ^year?

*How do they find the workload in the 3^rd ^year compared to yrs.1&2? What exactly is the problem, if any? Ask for examples*.

### Effect of exams on learning

How much of their learning is in response to exams? What is the effect of exams on their learning? What would happen to their learning if they didn't have exams?

Have they done really well in any exam/assessment? What was the effect of this on their learning?

### The ward learning environment

*Ask them to describe the good parts and the drawbacks*.

***Probe ****teaching by SHOs and PRHOs. Is what they learn on the wards easier to remember? Does the fact that it is in context help? How?*

### The informal peer group

Do they undertake any unofficial leaning with groups of friends? If so what form does it take? Is it helpful? How?
